# Ca^(2+)^N It Be Measured? Detection of Extramitochondrial Calcium Movement With High-Resolution FluoRespirometry

**DOI:** 10.1038/s41598-019-55618-5

**Published:** 2019-12-17

**Authors:** Anna Nászai, Emil Terhes, József Kaszaki, Mihály Boros, László Juhász

**Affiliations:** University of Szeged, Faculty of Medicine, Institute of Surgical Research, Szeged, Hungary

**Keywords:** Mitochondria, Calcium channels

## Abstract

Our aim was to develop a method to detect extramitochondrial Ca^2+^ movement and O_2_ fluxes simultaneously. Using High-Resolution FluoRespirometry, we also tested whether mitochondrial permeability transition pore (mPTP) inhibition or anoxia affects the mitochondrial Ca^2+^ flux. Ca^2+^ movement evoked by CaCl_2_ or anoxia was assessed with CaGreen-5N dye using Blue-Fluorescence-Sensor in isolated liver mitochondria, liver homogenates and duodenal biopsies. Exogenous CaCl_2_ (50 µM) resulted in an abrupt elevation in CaGreen-5N fluorescence followed by a decrease (Ca^2+^ uptake) with simultaneous elevation in O_2_ consumption in liver preparations. This was followed by a rapid increase in the fluorescence signal, reaching a higher intensity (Ca^2+^ efflux) than that of the initial CaCl_2_-induced elevation. Chelation of Ca^2+^ with EGTA completely abolished the fluorescence of the indicator. After pre-incubation with cyclosporin A, a marked delay in Ca^2+^ movement was observed, not only in isolated liver mitochondria, but also in tissue homogenates. In all samples, the transition to anoxia resulted in immediate increase in the level of extramitochondrial Ca^2+^. The results demonstrate that the CaGreen-5N method is suitable to monitor simultaneous O_2_ and Ca^2+^ fluxes, and the opening of mPTP in various biological samples. In this system the duration of stimulated Ca^2+^ fluxes may provide a novel parameter to evaluate the efficacy of mPTP blocker compounds.

## Introduction

Mitochondria are main controller units of calcium (Ca^2+^) homeostasis of the eukaryotic cell^1^. Various channels, transmembrane proteins and receptors have been identified in the regulation of mitochondrial Ca^2+^ influx, efflux and storage, and the net result of these processes fundamentally influences the activity of intracellular regulatory systems. Low micromolar concentration range (~0.1–10 µM) of Ca^2+^ activates various enzymes (e.g. piruvate dehydrogenase, isocitrate dehydrogenase and alpha-ketoglutarate dehydrogenase) in the mitochondrial matrix^2^, stimulates the outer mitochondrial membrane-bound monoamine oxidase A (MAO-A), the enzyme responsible for the degradation of biogenic amines^3^, and influences the action of the mitochondrial antioxidant system through the regulation of manganese superoxide dismutase (MnSOD) activity^4^. In this range, Ca^2+^ fine-tunes oxidative phosphorylation and ATP synthesis, and the electron transport system (ETS) can be either stimulated or depressed^2,5–7^. Higher concentrations (~50 µM) may, however, lead to the opening of the mitochondrial permeability transition pore (mPTP), with non-selective Ca^2+^ efflux, collapse of mitochondrial membrane potential and apoptosis-mediated cell death^8,9^. The multiple roles of Ca^2+^ in cellular and mitochondrial function and the mechanism of mPTP activation in various hypoxia-associated pathologies are in the focus of intense research interest and laboratory investigation^10^. However, despite its importance, current technical and analytic options have prevented the collection of essential data on simultaneous Ca^2+^ and O_2_ fluxes in cells or mitochondria^11,12^. Therefore, our aim was to design a method, where the respiratory chain activity and mPTP opening can be detected together with extramitochondrial Ca^2+^ concentrations. More directly, our aim was to examine the Ca^2+^-activated mPTP opening and mPTP-mediated Ca^2+^ release, in association with O_2_ consumption changes in isolated mitochondria, tissue homogenates and tissue biopsy samples. Since detailed protocols and technical information on the subject were not available, the effect of mPTP inhibition was also tested together with anoxia-induced changes in Ca^2+^ flux when the function of ETS was inhibited.

For this in-depth study, High-Resolution FluoRespirometry was used for the combined detection of Ca^2+^ and O_2_ fluxes, and Calcium Green-5N (CaGreen-5N), a single wavelength fluorescent dye, was employed to measure extramitochondrial Ca^2+^. It has been shown that this probe has a low affinity for Ca^2+^, making it suitable for evaluating relatively high Ca^2+^ concentrations (K_D_ 14000 nM, 0.5–50 µM) and insensitive to Mg^2+^ (Supplementary Fig. S1), which increases its selectivity^13^. Another advantage is that the Ca^2+^ signal remains stable for a longer period (at least for 120 min) with negligible bleaching and without shifting its excitation or emission wavelengths^14^.

## Materials and Methods

### Animals

Male Sprague–Dawley rats (330–360 g) and SKH-1 hairless mice (20–30 g) were used. The animals were housed in plastic cages (21–23 °C) with a 12/12 h dark-light cycle and *ad libitum* access to standard rodent chow and water. The experiments were performed in accordance with National Institutes of Health guidelines on the handling and care of experimental animals and EU Directive 2010/63 for the protection of animals used for scientific purposes. All animal experimental protocols were reviewed by the National Scientific Ethical Committee on Animal Experimentation (National Competent Authority of Hungary) and was approved by the Animal Welfare Committee of the University of Szeged (approval number V/175/2018). Tissue samples of liver and duodenum were taken after ketamine and xylazine (rats: 50 and 10 mg kg^−1^, mice: 80 and 24 mg kg^−1^ ip, respectively) anaesthesia.

### Reagents

CaGreen-5N (Hexapotassium Salt, cell impermeant) was purchased from Thermo Fisher Scientific (Waltham, Mass., USA). All other reagents, including respiratory substrates and inhibitors, were purchased from Sigma Aldrich (St. Louis, Mo., USA). Manual titration of these substances for 2 mL volume was carried out with Hamilton syringes. (Details on exact volumes and concentrations can be found at http://wiki.oroboros.at/images/f/fc/Gnaiger_2014_Mitochondr_Physiol_Network_MitoPathways.pdf).

### Composition of respiration media

In pilot experiments using liver samples and multicomponent MiR05 medium with or without EGTA (0.5 mM), Ca^2+^ fluxes were not detected. This could be attributed to the lactobionic acid, taurine and bovine serum albumin components of MiR05 that are suggested to bind/chelate Ca^2+^. For this reason, we used a mannitol- and sucrose-based respiration buffer^9^ in which Ca^2+^ fluxes were readily distinguished from addition of Ca^2+^ (Supplementary Fig. S2).

### Preparation of Ca^2+^ indicator

CaGreen-5N, a single wavelength fluorescent dye, was used to measure extramitochondrial Ca^2+^. This probe has a low affinity for Ca^2+^^13^, making it suitable for evaluating relative high Ca^2+^ concentrations (K_D_ 14000 nM, 0.5–50 µM). CaGreen-5N was dissolved through magnetic stirring in anhydrous dimethyl sulfoxide (DMSO), and 2 mM stock solution were prepared according to manufacture’s instructions. Stock solution was aliquoted (20 µL) in sterile Eppendorf vials, covered with strips of aluminium foil and stored at −20 °C until further use.

### The effect of respiratory substrates and inhibitors on CaGreen-5N fluorescence

Signal stability of CaGreen-5N was verified in 2 mL volume of respiration media after titration of substrates (10 mM glutamate, 2 mM malate, 10 mM succinate and 2.5 mM ADP) and inhibitors (0.5 µM rotenone, 2.5 µM antimycin A, 100 mM sodium azide and 1 µM cyclosporin A; Supplementary Fig. S3). Among these compounds, only the complex IV inhibitor sodium azide (NaN_3_) affected fluorescence markedly; here, a nearly 50% decrease in signal intensity was observed (Supplementary Fig. S3). This led us to avoid the use of NaN_3_ in optical measurements. Apart from the NaN_3_ effect, a chamber opening (removal of stopper) with a steep increase in the CaGreen-5N signal resulted in a fluorescent artefact as well (Supplementary Fig. 3).

### Calibration and measurements using High-Resolution FluoRespirometry

All mitochondrial measurements were performed using High-Resolution FluoRespirometry (O2k, Oroboros Instruments, Innsbruck, Austria). On the day of the experiment, a 40 min stabilization period was allowed for air calibration and temperature equilibration of the incubation medium, visualized as stabilization of the Peltier power (http://wiki.oroboros.at/images/7/77/MiPNet06.03_POS-Calibration-SOP.pdf). After 40 min, the O_2_ signals were stable with the O_2_ slope (uncorrected) close to zero (at gain 1 for sensor and 800 mV polarization voltage). Noise of the O_2_ slope was within ± 2 pmol s^−1^ mL^−1^ at a data recording interval of 2 sec and 40 data points selected for calculation of the slope. Calibration and measurements were performed during continuous stirring (750 rpm) at 37 °C in a 2 mL respiration medium^9^ containing 210 mM mannitol, 70 mM sucrose, 0.2 mM KH_2_PO_4_ and 5 mM Tris-HCl adjusted to pH 7.4. The DatLab software (Oroboros Instruments, Innsbruck, Austria) was used for online display, FluoRespirometry data acquisition and analysis. Blue Fluorescence-Sensor (excitation 465 nm; gain for sensor: 1000 and polarization voltage: 500 mV) was connected^15^ to the windows on the glass chambers and the instrument (Oroboros Instruments, Innsbruck, Austria) to measure flourescence. Ca^2+^-related changes were expressed as the rate of changes in fluorescent signal and average resting fluorescence using the following formula: ∆Ca^2+^ = ∆*F*/*F* = (*F* *−* *F*_*rest*_)/*F*_*rest*_, where *F* is the indicator fluorescence at any given time during the experiments and *F*_*rest*_ is the average fluorescence signal before treatment (e.g. exogenous Ca^2+^) or before the start of *in vitro* anoxia in the respiration chamber.

### MPTP-mediated Ca^2+^release in isolated mitochondria

**Figure 1:**
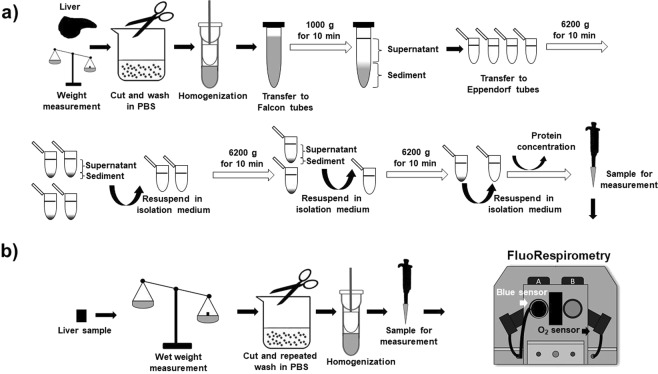
Preparation of isolated liver mitochondria and liver homogenate. The whole liver was harvested to prepare isolated mitochondrial fraction (**a**) or the left lateral liver lobe (**b**) was used for homogenate preparation. After differential centrifugation or homogenization, a High-Resolution FluoRespirometer equipped with Fluorescence Sensor Blue was used for functional measurements.

A modified method described by Sumbalova *et al*. (2016) was used for mitochondria isolation (See http://wiki.oroboros.at/images/d/dc/MiPNet20.08_IsolationRatLiver-mt.pdf). The liver tissue was dissected and placed in ice-cold phosphate buffered saline (PBS; pH 7.4), and wet weight was measured (1.5 g of liver tissue was used for isolation). Liver samples were cut into small pieces with a sharp scissors, suspended in 10 volumes of ice-cold isolation medium (225 mM mannitol, 5 mM sucrose and 0.2 mM EDTA adjusted pH to 7.4) and transferred to a pre-cooled Potter-Elvehjem. After homogenization (10 strokes), samples were centrifuged at 1000 g for 10 min at 4 °C (Fig. 1a). Then 1.2 mL supernatant was transferred to 1.5 mL Eppendorf tubes and centrifuged at 6200 g for 10 min at 4 °C. After the second centrifugation, the supernatant was discarded, and sediment containing mitochondria was resuspended in 600 µL isolation medium each. Samples in 1.2 mL (2 resuspended Eppendorf tubes) isolation medium were centrifuged again at 6200 g for 10 min at 4 °C. Finally, the supernatant was discarded, mitochondria was resuspended in 100 µL isolation medium and stored on ice for no more than 3 h until measurement. Protein content was determined with the Biuret method from fresh mitochondrial samples, and 0.15 mg mL^−1^ concentration was used for the FluoRespirometric measurements. Thirty min after isolation, the succinate pathway control state (http://www.bioblast.at/index.php/Succinate_pathway_control_state) was activated with complex II substrate (S; 10 mM succinate). To prevent accumulation of oxaloacetate (a known endogenous inhibor of succinate dehydrogenase), complex I was blocked with 0.5 µM rotenone (Rot) prior to succinate administration. Stimulation of oxidative phosphorylation (OXPHOS) with adenosine diphosphate (ADP) was omitted from the protocol due to its inhibitory effect on mPTP opening^16^. After stable respiration, Ca^2+^ movement was assessed with CaGreen-5N fluorescent dye (2 µM; excitation: 506 nm; emission: 532 nm). Mitochondrial Ca^2+^ influx and subsequent mPTP-mediated Ca^2+^ efflux were stimulated with the addition of 50 µM calcium chloride (CaCl_2_). When maximum fluorescence was reached, 1 mM EGTA was used for Ca^2+^ removal. ETS-independent respiration (or residual O_2_ consumption; ROX) was determined after complex III inhibition with antimycin A (AmA; 2.5 µM). Duration of Ca^2+^ fluxes (*t1* influx and *t2* efflux) was expressed in seconds (s), whereas volume-specific O_2_ flux (J_V,O2_) was expressed in pmol sec^−1^ mL^−1^.

### MPTP-mediated Ca^2+^ release in tissue homogenate

The experiments were expanded to determine the simultaneous Ca^2+^ flux and O_2_ consumption in homogenate samples (Fig. 1b). A modified fluorescent method developed by Elustondo *et al*. in isolated liver mitochondria^9^ was used to measure homogenate Ca^2+^ flux. In brief, tissue biopsies were obtained from the left lateral liver lobe (~300 mg), cut into small (<20 mg) pieces, washed five times with PBS (pH 7.4) and then homogenized (with a Potter-Elvehjem, 1:10% w V^−1^) in isolation media containing 225 mM mannitol, 5 mM sucrose and 0.2 mM EDTA (pH 7.4). All the mitochondrial samples were energized with complex II substrate (10 mM succinate) after complex I blockade with rotenone (0.5 µM). Stimulation with ADP was omitted from the protocol due to its inhibitory effect on mPTP opening^16^. After reaching a stable respiration, Ca^2+^ movement was assessed with 2 µM CaGreen-5N fluorophore. Ca^2+^-induced Ca^2+^ flux was stimulated with the addition of 50 µM CaCl_2_. At the end of mPTP-mediated Ca^2+^ release, 1 mM EGTA was used to chelate Ca^2+^. ROX was determined after complex III inhibition with antimycin A (2.5 µM). Duration of Ca^2+^ signals (*t1* and *t2*) was expressed in seconds (s), whereas volume specific O_2_ flux (J_V,O2_) was expressed in pmol sec^−1^ mL^−1^ normalized to 8 mg wet weight per chamber.

### Ca^2+^ movement in isolated mitochondria under anoxia

To elucidate the mechanism of transition from hypoxia to anoxia (anoxia was defined as ~0 nmol mL^−1^ O_2_ concentration in O2k chambers at 37 °C), samples were allowed to consume dissolved O_2_ available in respiration media. Thus, the chambers were kept closed throughout the experiments (to avoid any contact with air under anoxia), and oxygenation (e.g. O_2_ generation by catalase from hydrogen peroxide) or addition of O_2_ (e.g. O_2_ gas mixture injected through a needle) was avoided through the central capillary^17^. Black cover-slips were placed on top of stoppers to prevent light penetrating the capillary and avoid disruption of the fluorescence signal. The preparation protocol, instrument set-up and respiration media were identical to those for mitochondria exposed to CaCl_2_, as described above. Samples were treated with 0.5 µM rotenone, 10 mM succinate, 2.5 mM ADP and 2 µM CaGreen-5N before anoxia. After anoxia, responsiveness of fluorescent dye was monitored by exogenous 15 µM CaCl_2._ The Ca^2+^ signal was abolished with the addition of 1 mM EGTA.

### Ca^2+^ movement in homogenate under *in vitro* anoxia

Anoxic Ca^2+^ release was assessed in liver homogenate as well. Preparation of homogenate and anoxia induction were identical as described above. Homogenate samples were treated with 0.5 µM rotenone, 10 mM succinate, 2.5 mM ADP and 2 µM CaGreen-5N. After anoxia, sensitivity of fluorophore was tested with 15 µM CaCl_2_. Ca^2+^ in the respiration medium was chelated with 1 mM EGTA.

### Monitoring Ca^2+^ fluxes in intestinal biopsy samples

**Figure 2:**
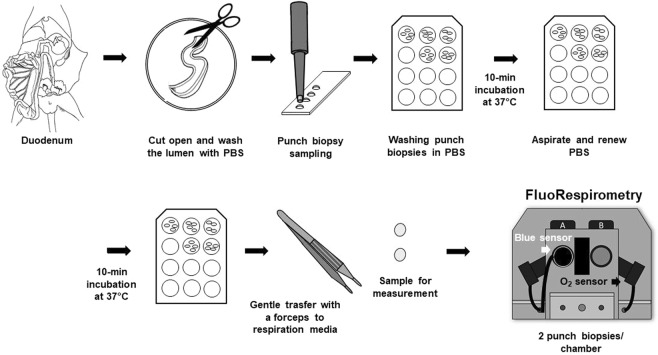
Preparation of punch biopsies. An intestinal section was harvested from mouse duodenum. After the lumen was opened, faeces was removed with a PBS flush. Punch biopsy (4 mm Stiefel) was used to cut tissue disks from the intestines. The circular biopsies were put into cell culture plates (4/well) containing 400 µL PBS and incubated at 37 °C for 2 × 10 mins. Two punch biopsies were transferred gently into respiration media and used for FluoRespirometric measurements.

Mouse doudenal samples were used to detect Ca^2+^ movement in tissue biopsies. Previous data have shown that samples from this site exhibit stable ROUTINE respiration (i.e. “respiration without external substrate and ADP”) and that they are responsive to ATP synthase inhibitor (2.5 µM oligomycin), as compared to other intestinal segments (Supplementary Fig. S4). In brief, an approximately 3 cm-long duodenal segment was removed (Fig. 2) and immediately placed in PBS (pH 7.4). The luminal content was rinsed three times with PBS solution using a 1 mL syringe. The empty duodenum was cut open, gently placed in a covered glass Petri dish kept on ice with ophthalmic forceps. The intestinal samples were covered with a Foliodrape drape sheet and PBS solution to prevent drying. A 4 mm diameter disposable punch biopsy knife (Integra Miltex) was used, and the biopsies were immediately transferred to a 12-well plate (Costar) washed with 400 µL PBS for 10 min at 37 °C. Measurements were performed in 2 mL of mannitol- and sucrose-based medium at 37 °C. Two punch biopsies per respiratory chamber were energized with succinate (S; 10 mM in the presence of rotenone; exogenous Ca^2+^) or with succinate and 2.5 mM ADP (Rotenone + S + ADP; estimation of endogenous Ca^2+^) and used for analysis. Exogenous CaCl_2_-induced and anoxia-induced Ca^2+^ fluxes were determined from these samples in the presence of 2 µM CaGreen-5N using Fluorescence-Sensor Blue. Finally, 1 mM EGTA was injected into the chambers for the chelation of Ca^2+^. The instrumental set-up, including excitation, gain and polarization voltage, was identical with the set-up used for isolated mitochondria and homogenate.

### Modulation of Ca^2+^ fluxes through mPTP inhibition

We used isolated mitochondria and homogenate for the pharmacological modulation of extramitochondrial Ca^2+^ flux. In the experiment setting, non-specific Ca^2+^ release through mPTP was inhibited by 1 µM cyclosporin A. The inhibitor was pipetted to energized mitochondria (rotenone + succinate), and then samples were incubated for 3 min at 37 °C. After chamber closure, Ca^2+^ movement was stimulated with 50 µM CaCl_2_, and Ca^2+^ signal was abolished with 1 mM EGTA, according to the previously described protocol.

### Statistical analysis

All data were expressed as mean ± SD; differences between means were compared using Student’s *t* test and ANOVA for repeated measures using Fisher’s LSD post hoc test, as appropriate. Data analysis was performed with a statistical software package (SigmaStat for Windows, Jandel Scientific, Erkrath, Germany). A value of *P* < 0.05 was considered statistically significant.

## Results

### Exogenous Ca^2+^-induced Ca^2+^ flux and the effect of anoxia in isolated liver mitochondria

**Figure 3:**
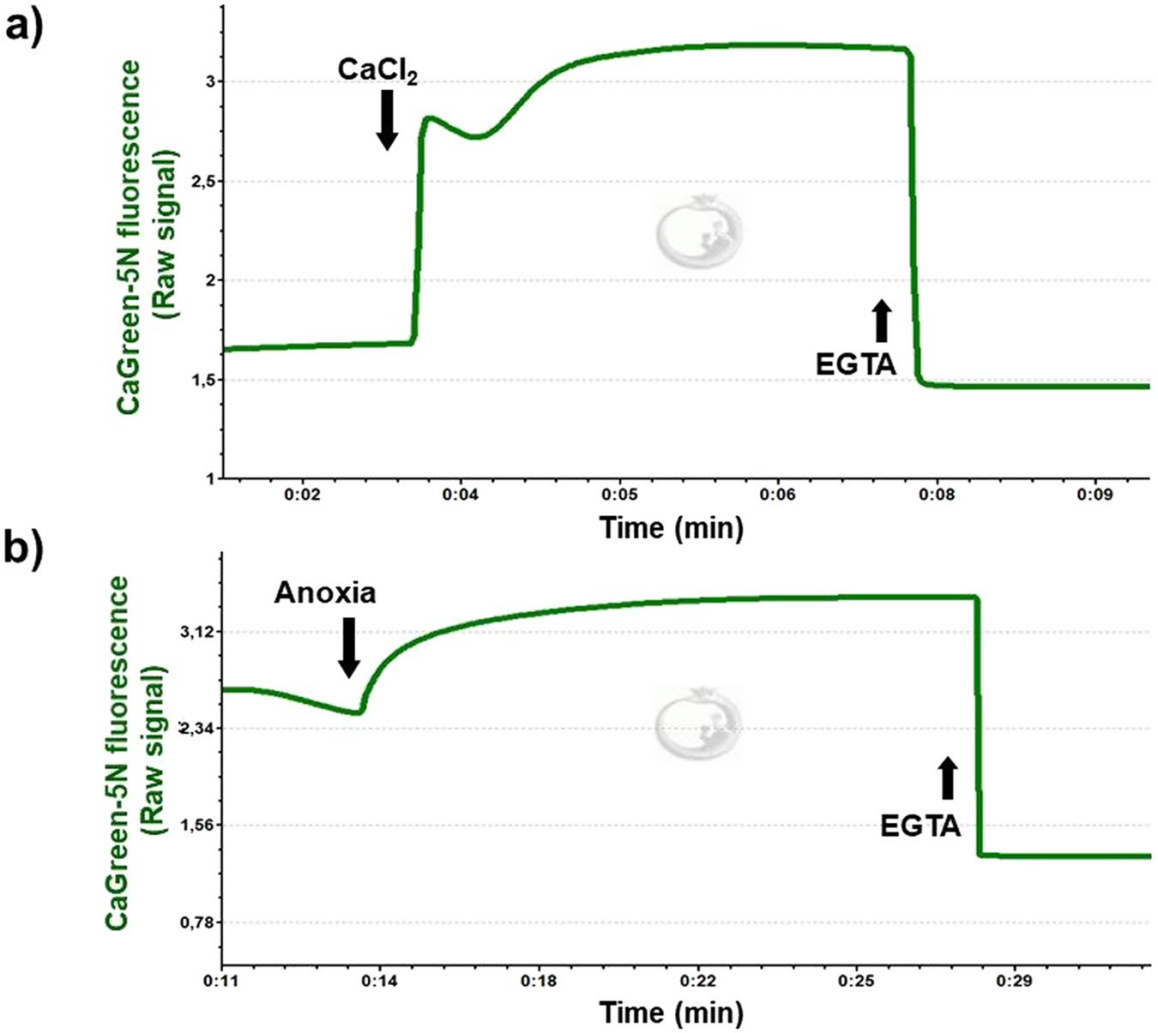
Addition of exogenous Ca^2+^ and anoxia-mediated changes in Ca^2+^ flux. Mitochondrial Ca^2+^ fluxes were induced by a single administration of 50 µM CaCl_2_ (**a**) or released endogenously (**b**) during anoxia. Signals were abolished with 1 mM EGTA.

**Figure 4:**
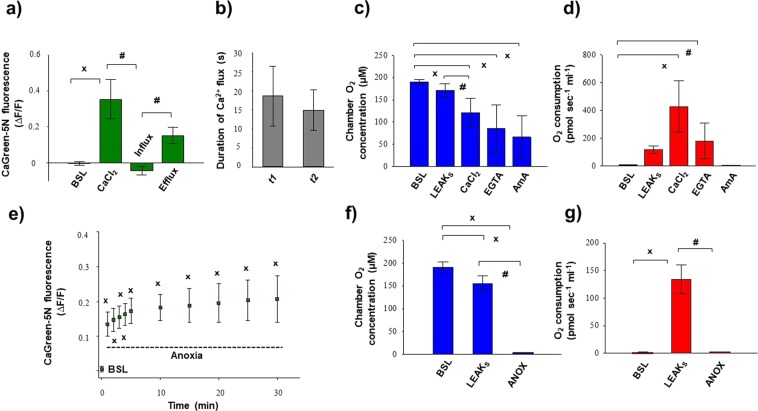
Exogenous Ca^2+^- (**a**–**d**) and anoxia- (**e–g**) induced changes in Ca^2+^ flux, O_2_ flux and O_2_ concentration in isolated liver mitochondria. An immediate increase in Ca^2+^ fluorescence was found both after the injection of CaCl_2_ and commencement of anoxia. Bars (**a**–**g**) show means ± SD, n = 4 independent rats measured in duplicate (**a**–**d**) and n = 5 independent rats (**e**–**g**). ^X^*P < *0.05 vs before anoxia or Ca^2+^ stimulus (BSL) and ^#^*P* < 0.05 vs Ca^2+^ influx, anoxia and LEAKs.

Representative Ca^2+^ fluxes registered in isolated rat mitochondria are shown in Fig. 3. After a stable fluorescent signal, the mitochondrial Ca^2+^ flux was stimulated with 50 µM exogenous CaCl_2_ (Fig. 3a), resulting in an immediate elevation in fluorescent intensity. A few seconds later, a decline in the CaGreen-5N signal and a simultaneous increase in O_2_ consumption were observed, indicating the uptake of external Ca^2+^ (Fig. 4a–d). When minimal fluorescence was reached, mitochondria started to remove Ca^2+^ and fluorescent intensity increased again, reaching a higher value than that of the initial baseline fluorescence. This increase was most probably due to the non-selective Ca^2+^ efflux through mPTP^9^. Figure 3b shows the effect of anoxia on CaGreen-5N fluorescence. The transition to anoxia in isolated mitochondria itself provoked an immediate increase in Ca^2+^ efflux (Fig. 3b). A common property of fluxes is that the Ca^2+^ chelator EGTA completely abolishes the signal within 3 seconds, no matter whether Ca^2+^ flux was stimulated exogenously or mediated endogenously during anoxia.

Figure 4a–d summarises the changes in Ca^2+^ flux, O_2_ flux and O_2_ concentration in mitochondria induced by the addition of exogenous Ca^2+^. The duration of Ca^2+^ fluxes (influx and efflux) was similar, not exceeding 30 seconds. Simultaneously with these fluxes, a marked elevation in O_2_ consumption was found after addition of Ca^2+^. Non-electron transport system-derived respiration was negligible in our preparations, since residual O_2_ consumption was close to zero (tested with AmA; Fig. 4d). A continuous decrease in chamber O_2_ concentration was obvious, but there was no limitation (it remained above 40 µM), particularly during stimulation with exogenous Ca^2+^.

Figure 4e illustrates the anoxia-induced changes in the levels of Ca^2+^ that were evaluated at zero O_2_ flux and O_2_ consumption (Fig. 4f,g). The transition to anoxia immediately increased Ca^2+^ efflux, which became statistically significant 1 min after complete O_2_ restriction and remained elevated during the experiments.

### Exogenous Ca^2+^-induced Ca^2+^ flux and the effect of anoxia in liver homogenate

**Figure 5:**
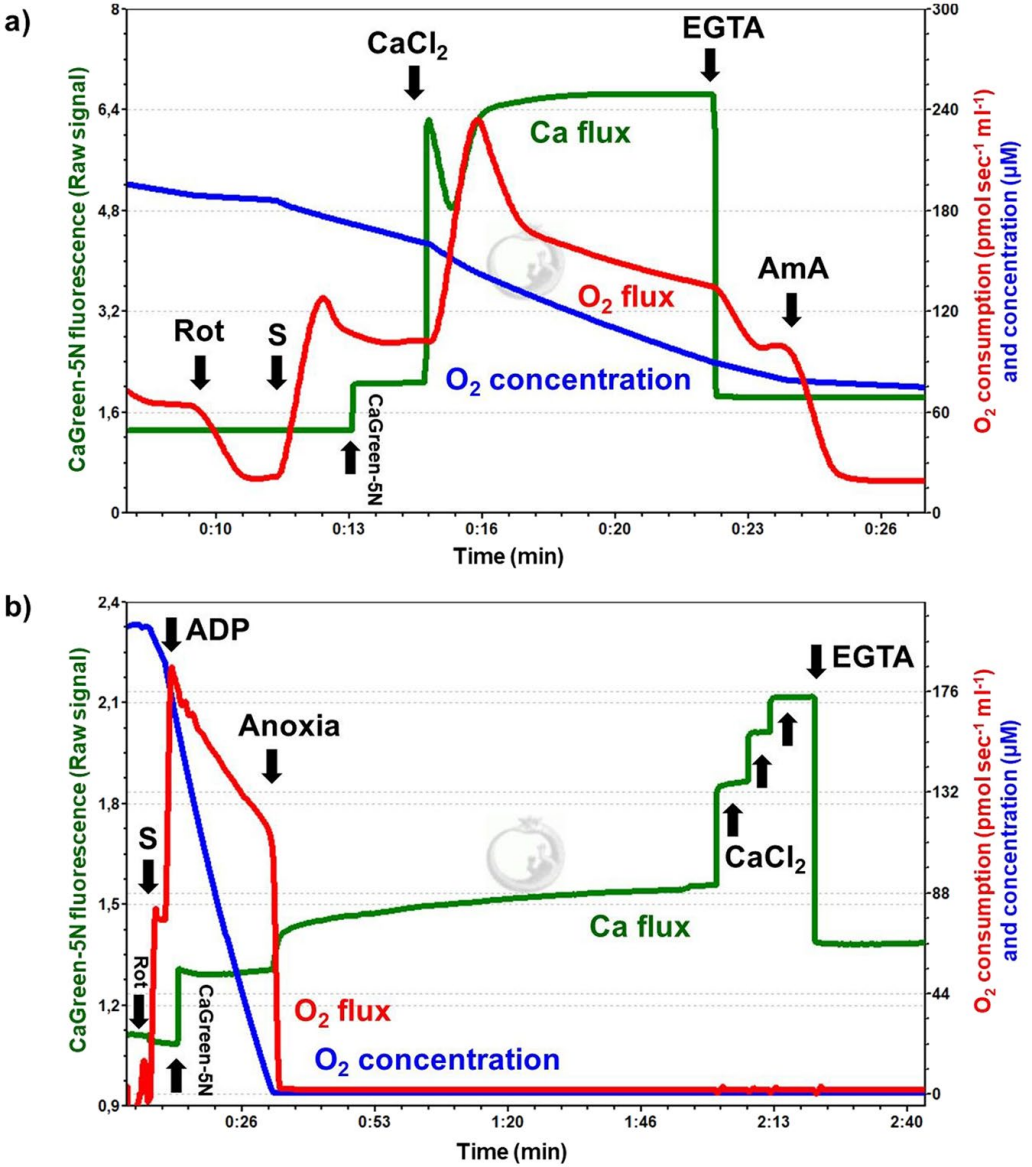
Simultaneous measurement of Ca^2+^ and O_2_ flux after exogenous Ca^2+^ administration (**a**) and during anoxia (**b**). Superimposed lines illustrate extramitochondrial Ca^2+^ flux (green), mitochondrial O_2_ consumption (O_2_ flux; red) and O_2_ concentration (blue). Mitochondrial Ca^2+^ fluxes were induced by a single administration of 50 µM CaCl_2_ (**a**). Sensitivity of fluorophore during anoxia was tested by repeated addition of 15 µM CaCl_2_ (**b**).

Representative registration of Ca^2+^ and O_2_ flux acquired by O2k-FluoRespirometer is shown in Fig. 5. The CaGreen-5N indicator was injected after activation of the succinate pathway control state. After the stabilization of respiration and the fluorescent signal, mitochondrial Ca^2+^ flux was stimulated with the addition of 50 µM CaCl_2_ (Fig. 5a), resulting in an immediate increase in fluorescence. Then a few seconds later, a decline in the CaGreen-5N signal and a simultaneous increase in O_2_ consumption were observed, indicating an uptake of external Ca^2+^. Soon after minimal fluorescence was reached, mitochondria pumped Ca^2+^ out and fluorescent intensity started to elevate again, reaching a higher value than the initial baseline intensity. A decline in respiration (I) and completion of fluxes (II) are indicators of mPTP activation.

**Figure 6:**
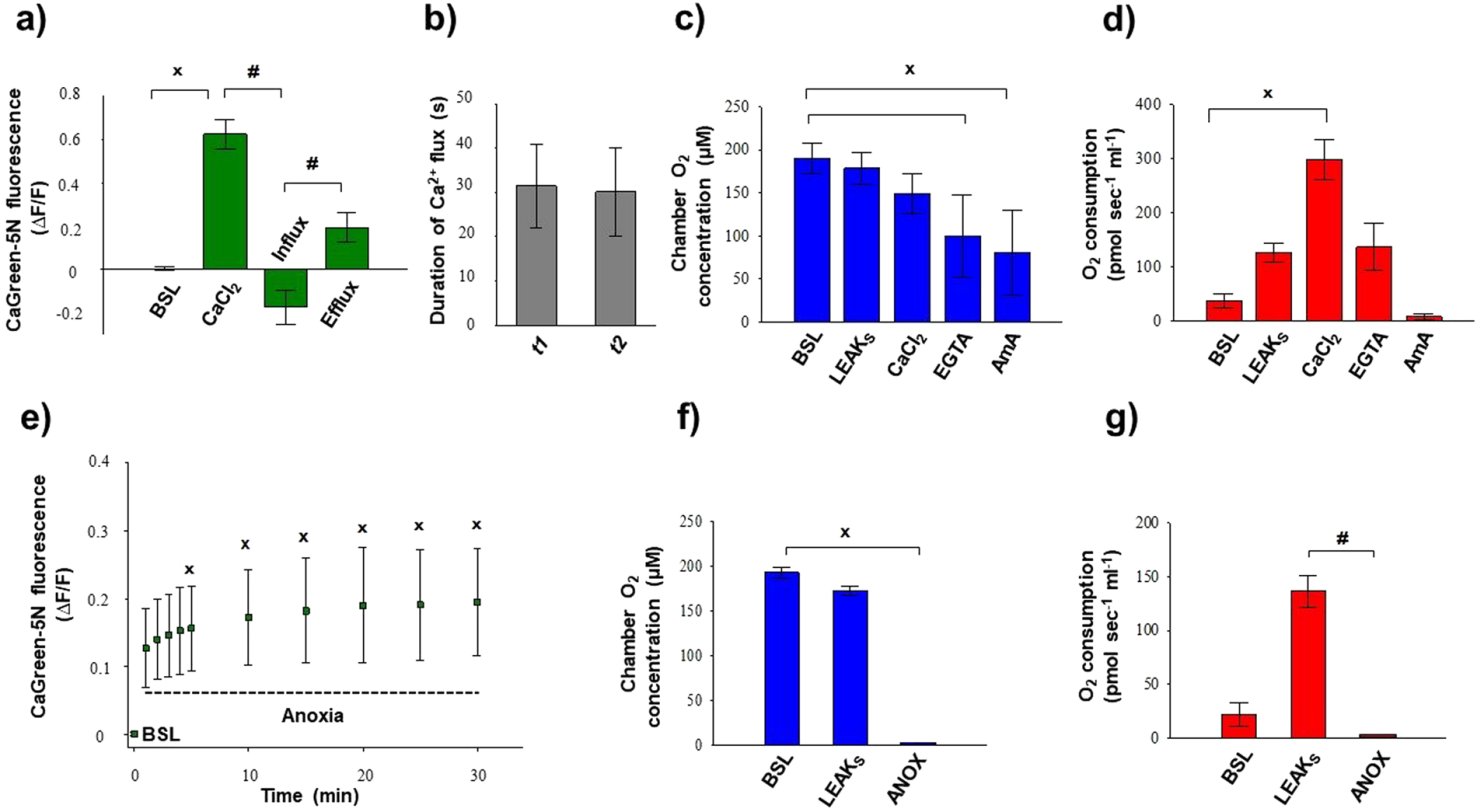
Exogenous Ca^2+^- (**a**–**d**) and anoxia- (**e**–**g**) induced changes in Ca^2+^ flux, O_2_ flux and O_2_ concentration in liver homogenate. An immediate increase in Ca^2+^ fluorescence was found both after the injection of CaCl_2_ and anoxia. Bars (**a**–**g**) show means ± SD, n = 8 independent rats. ^X^*P < *0.05 vs before anoxia or Ca^2+^ stimulus (BSL) and ^#^*P* < 0.05 vs Ca^2+^ influx and anoxia.

Figure 6 shows the external Ca^2+^-evoked quantitative changes in Ca^2+^ flux, O_2_ consumption and O_2_ concentration in rat liver homogenate. In each sample, the duration of both Ca^2+^ influx and efflux was fast, with a maximum value of ~40 s (Fig. 6b). A prompt increase in mitochondrial O_2_ consumption was detected after the addition of Ca^2+^, which started to decline soon after the Ca^2+^-induced O_2_ peak. EGTA chelated extramitochondrial Ca^2+^ and a similar O_2_ flux to succinate supported respiration (LEAK_S_) were recorded (Fig. 6d). The complex III inhibitor antimycin A almost completely abolished the electron transport system-dependent respiration (or ROX). The O_2_ concentration in the chamber remained above 50–100 µM after Ca^2+^ injection; thus, there was no limitation of O_2_ for CaCl_2_-induced respiratory stimulation (Fig. 6c).

Anoxia was confirmed by measuring zero O_2_ flux and O_2_ consumption in respiration chambers, which are illustrated in Figs. 5b, 6f,g. Prior to anoxia (Fig. 6e, BSL), fluorescence was not changed markedly, but transition from normoxia to anoxia elevated the CaGreen-5N signal. Ca^2+^ release became statistically significant 5 min after the commencement of anoxia and remained elevated for a 30 min period (Fig. 6e). As with the addition of exogenous CaCl_2_, 1 mM EGTA blocked release of endogenous Ca^2+^ under anoxia and completely abolished the Ca^2+^ signal.

### FluoRespirometry with duodenal punch biopsies

**Figure 7:**
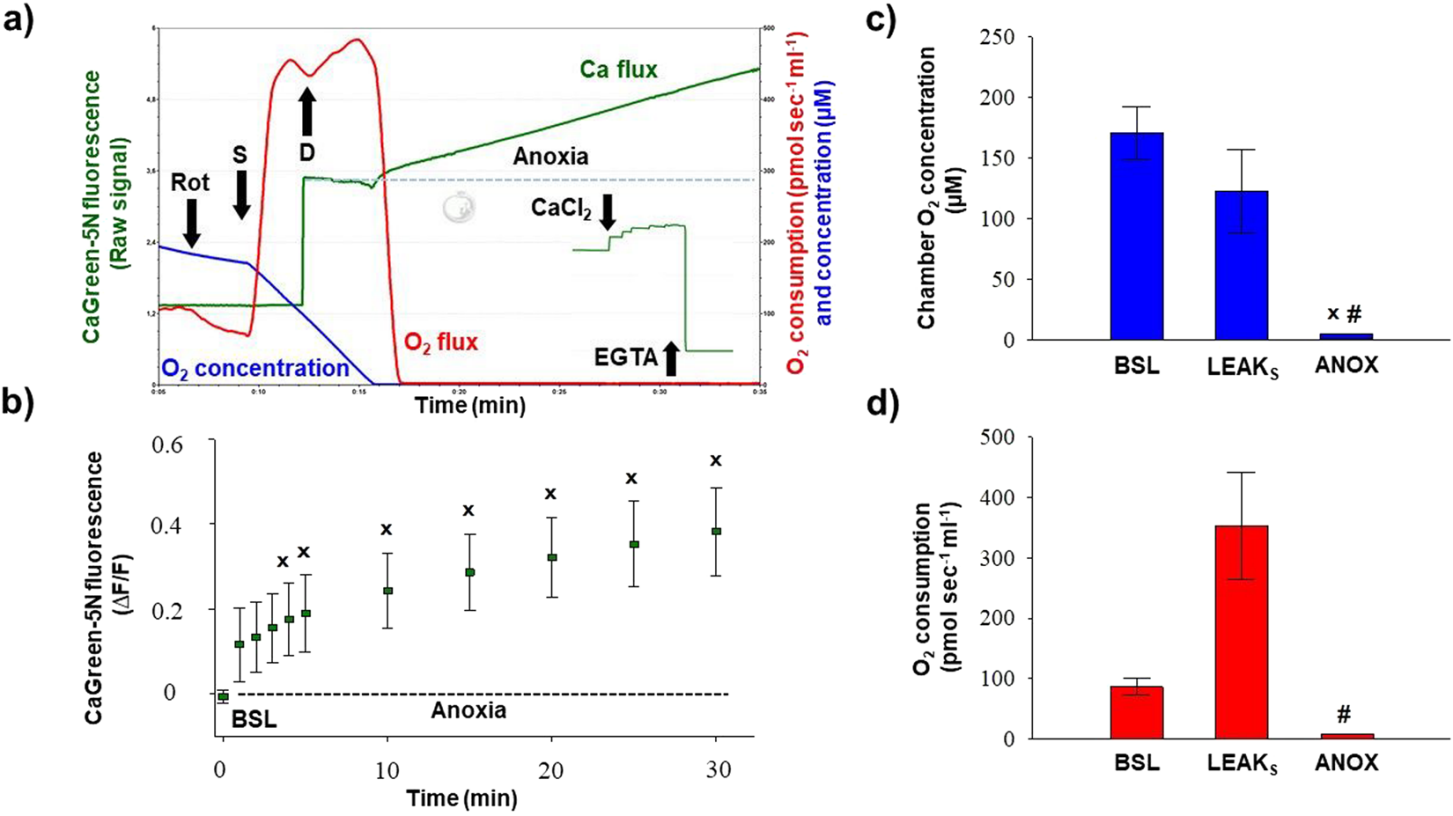
Anoxic transition in duodenal punch biopsies. Anoxia (ANOX) resulted in an immediate increase in Ca^2+^ fluorescence. Responsivness of fluorophore under anoxia was checked by adding CaCl_2_ (15 µM) and 1 mM EGTA. Superimposed lines in (**a**) illustrate Ca^2+^ flux (green), O_2_ consumption (O_2_ flux; red) and O_2_ concentration (blue) in the respiration chamber. Bars (**b**–**d**) show means ± SD, n = 4 independent mice measured in duplicate. ^X^*P < *0.05 vs before anoxia (BSL) and ^#^*P* < 0.05 vs LEAK_S_.

Figure 7 shows the simultaneous changes in Ca^2+^ flux, O_2_ concentration and O_2_ consumption measured in duodenal samples. Punch biopsies consumed all dissolved O_2_ in the respiration medium, resulting in zero chamber O_2_ concentration and O_2_ flux (Fig. 7c,d). Our FluoRespirometric registration indicates (Fig. 7a) that in mannitol- and sucrose-based media, the previously found high capacity of oxidative phosporylation (which was assessed in a Mir05 medium) was absent, with only a small increase in O_2_ flux having been found. Also, the exogenous Ca^2+^-induced elevation in O_2_ consumption was not detected (data not shown). However, the inhibitory effect of rotenone and stimulatory effect of succinate were manifested on these duodenal segments. The CaGreen-5N signal was stabilized before anoxia; however, anoxic transition immediately increased its fluorescence, and this increase remained continuous more than 30 min throughout the measurement (Fig. 7a,b). The dye exhibited changes to a low micromolar Ca^2+^ (15 µM) and EGTA (1 mM) under anoxia (Fig. 7a) as well.

### The effect of mPTP inhibition on Ca^2+^-induced Ca^2+^ fluxes

**Figure 8:**
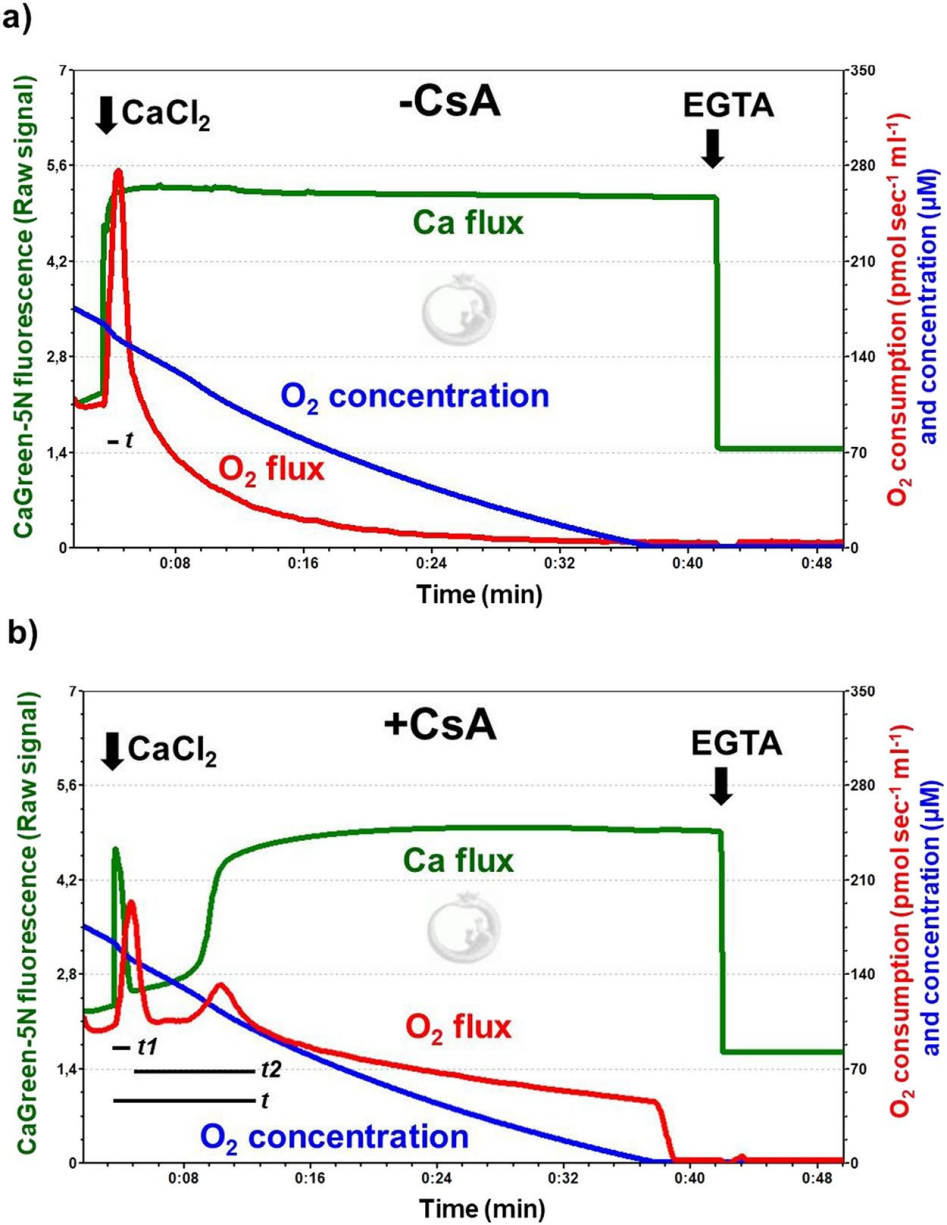
Simultaneous measurement of mitochondrial Ca^2+^ and O_2_ flux in the presence and absence of cyclosporin A. Inhibition of mPTPs with cyclosporin A (CsA) elevated Ca^2+^ uptake and delayed Ca^2+^ efflux after stimulation with exogenous Ca^2+^ (**b**). Superimposed lines illustrate extramitochondrial Ca^2+^ flux (green), mitochondrial O_2_ consumption (O_2_ flux; red) and O_2_ concentration (blue). Black lines illustrate the duration of Ca^2+^ fluxes (*t*), and the component influx (*t1*) and efflux (*t2*).

**Figure 9:**
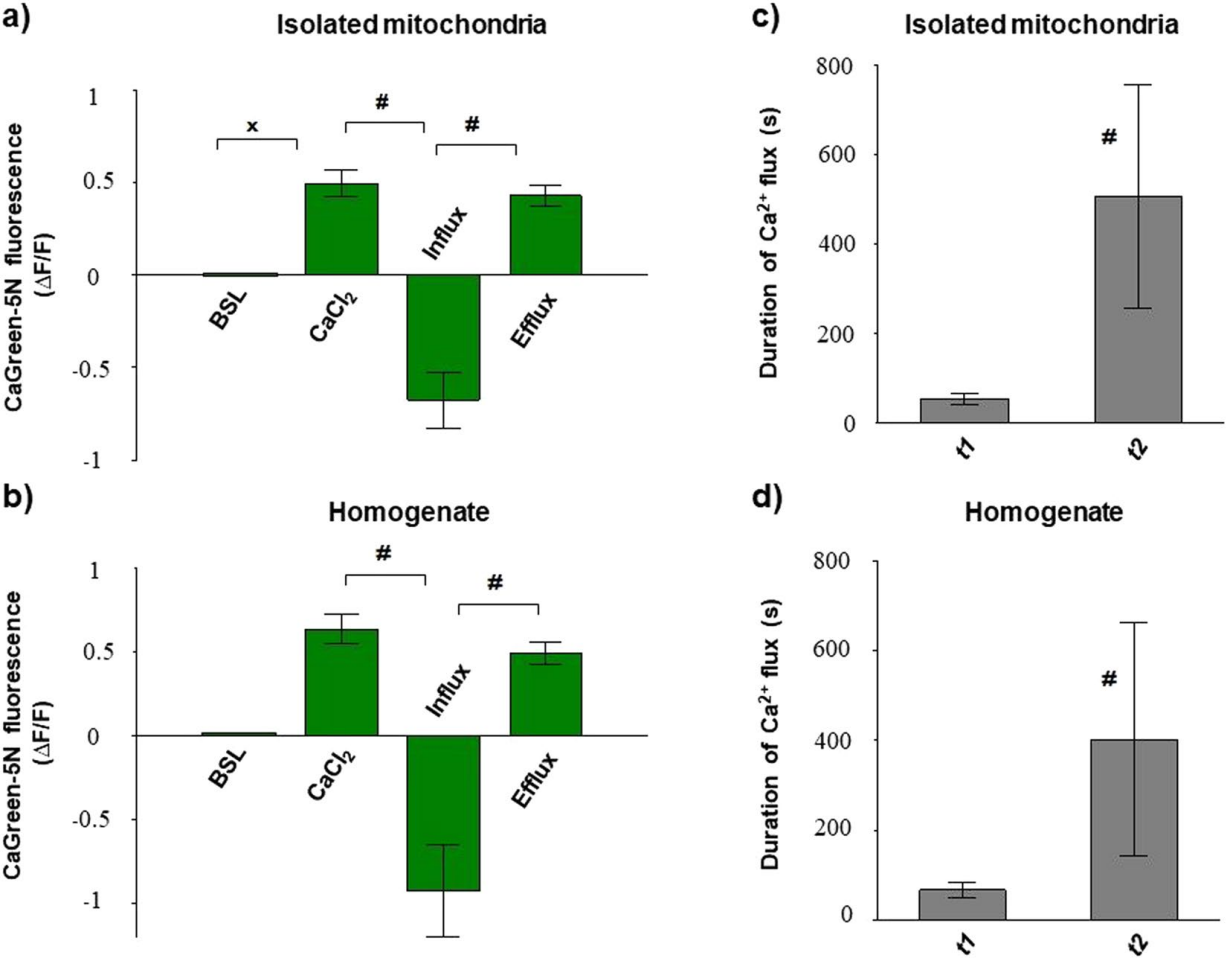
Ca^2+^ flux after mPTP inhibition in rat isolated mitochondria and liver homogenate. Changes in CaGreen-5N fluorescence (**a**,**b**) and duration of stimulated Ca^2+^ fluxes (**c**,**d**) are illustrated. Bars show means ± SD, n = 5 rats. ^X^*P < *0.05 vs before Ca^2+^ stimulus (BSL) and ^#^*P* < 0.05 compared to fluorescent intensity of Ca^2+^ influx or *t1*.

DatLab registrations in Fig. 8 illustrate simultaneous measurement of mitochondrial Ca^2+^ and O_2_ flux in the absence (Fig. 8a) and presence (Fig. 8b) of CsA. Pre-incubation with the inhibitordelayed Ca^2+^ flux, particularly the duration of Ca^2+^ efflux (*t2*), was indicated by slow elevation of fluorescence after the maximum Ca^2+^ uptake. Secondly, Ca^2+^ influx was also increased, since a more pronounced decrease in CaGreen-5N signal was found after stimulation. Third, the Ca^2+^-induced respiratory stimulation occurred in a different manner. In contrast with the vehicle-treated mitochondria, where a single injection of CaCl_2_ resulted in a single peak in O_2_ consumption, CsA pretreatment resulted in two well-distinguishable O_2_ peaks or a delayed peak in maximum O_2_ consumption. Figure 9 summarises the fluorescent changes and the duration of Ca^2+^ fluxes in rat isolated mitochondria and liver homogenate. The duration of Ca^2+^ uptake and that of subsequent Ca^2+^ release were very similar (*t1*: 19 ± 8 and *t2*: 15 ± 5 in isolated mitochondria; *t1*: 32 ± 9 and *t2*: 30 ± 10 in homogenate) without CsA, whereas in the presence of CsA, a delayed Ca^2+^ efflux and distinct t1/t2 rate were observed (*t1*: 53 ± 14 and *t2*: 506 ± 250 in isolated mitochondria and *t1*: 66 ± 17 and *t2*: 402 ± 259 in homogenate) both in rat isolated mitochondria and homogenate (Fig. 9c,d). This delayed effect was associated with a more pronounced decrease in fluorescence after 50 µM of CaCl_2_ injection. Similar results were obtained from SKH-1 mice (Supplementary Fig. S5).

## Discussion

Our study describes a novel method to detect simultaneous Ca^2+^ and O_2_ fluxes in various biological samples, together with exogenous Ca^2+^-induced mPTP opening and anoxia-induced changes in Ca^2+^ level. We employed High-Resolution FluoRespirometry, an established method for the simultaneous evaluation of mitochondrial functional indices and respiration^15,18,19^, and we combined the O_2_ and Ca^2+^ flux measurements using CaGreen-5N, a probe that reversibly binds to Ca^2+^^12^.

In the first set of experiments, liver mitochondrial preparations and intestinal biopsies were kept under normoxic conditions (O_2_ concentration was above ~150 µM before the addition of higher concentrations of CaCl_2_), and external Ca^2+^ was added to activate mPTPs and also to promote non-selective Ca^2+^ release through these channels. Both isolated mitochondria and tissue homogenate were responsive to a higher exogenous Ca^2+^ (50 µM), whereas the duodenal biopsies did not exhibit marked changes in CaGreen-5N fluorescence and increase of O_2_ consumption after the same insult. The reason behind the tissue-specific differences may stem from the more complex cellular milieu of intestinal punch biopsies. Duodenal segments contain various type of cells from all layers of bowel. It may well be that a higher external Ca^2+^ concentration should be used for the induction of Ca^2+^ fluxes. Secondly, we have no information on the magnitude of cellular Ca^2+^ influx (whether Ca^2+^ uptake actually occurs) and circumstances that may potentially contribute to elimination of Ca^2+^ (binding of Ca^2+^ by membrane proteins, lipids and other endogenous chelators) or modulate Ca^2+^ homeostasis (ER-mediated Ca^2+^ release in duodenal muscle layers).

Stimulated fluxes in liver-isolated mitochondria and homogenate, however, exhibited similar Ca^2+^ signals. Shortly after CaCl_2_ administration, a reduction of CaGreen-5N signal and a simultaneous increase in O_2_ consumption were observed, indicating an uptake of external Ca^2+^. Elevated O_2_ flux was also detected in these preparations when mitochondria started to pump Ca^2+^ out and therefore the fluorescence signal increased again. Another common point is that fluorescent intensity reached a higher value than that of the initial maximum Ca^2+^ peak after a single addition of CaCl_2_, indicating a massive Ca^2+^ efflux through mPTPs. Furthermore, the opening of these channels (reaching the fluorescence maximum) was accompanied by a decline in respiration. Of note, EGTA abolished the signal within 3 sec independently of the type of sample. The duration and rate of Ca^2+^ influx and subsequent Ca^2+^ efflux were also very similar in isolated mitochondria and homogenate in the absence of the mPTP inhibitor. When the samples were treated with CsA, the duration of stimulated Ca^2+^ fluxes, in particular Ca^2+^ efflux (*t2*), was prolonged, the Ca^2+^ influx was elevated (indicated by an abrupt decline in fluorescence), and dyscoupled respiration was delayed, indicated either by two O_2_ peaks or a single, delayed peak in maximum O_2_ consumption. The prolonged duration of mitochondrial dyscoupling (indicated by an increase in J_V,O2_ after 50 µM CaCl_2_) may be associated with a delayed inner membrane depolarization and inhibiton of membrane potential disruption.

To our best knowledge, these are the first results to describe and characterize simultaneous changes in Ca^2+^ and O_2_ flux mediated by mPTP inhibition, both in isolated mitochondria and tissue homogenate. In fact, fluorescent techniques are available to determine mPTP opening, including spectrofluorimetric determination of Ca^2+^ retention capacity in isolated mitochondria (CRC^11,12^), fluorescence microscopy protocols in living cells using the calcein-cobalt technique or mitochondrial membrane potential changes (TMRM) and ionomycin-induced swelling and mitochondrial network fragmentation assay with mitochondrially-targeted GFP^20^. However, all these methods focus on immediate changes in a single mitochondrial parameter. In addition, the function of ETS is critically dependent on the O_2_ concentration dissolved in a medium and the O_2_ availability at the surface of the sample. The limitation of O_2_ is a significant shortcoming of all in *in vitro* studies with mitochondrial preparations or cells because the insufficient O_2_ decreases the maximum capacity of OXPHOS and decreases the uncoupler- or Ca^2+^-triggered respiratory stimulation as well.

FluoRespirometric measurements with CaGreen-5N have previously been reported^9^, but several challenging questions have remained unanswered. In this respect, our most important technical modifications were as follows: 37 °C was used instead of 25 °C, the succinate pathway control state was activated instead of the NADH electron transfer pathway state, the composition of the medium used for isolation was improved, and the fluorophore tracer concentration was increased (from 1 µM to 2 µM). In pilot studies, we ascertained that these changes will make the detection of exogenous Ca^2+^-triggered Ca^2+^ fluxes possible. Based on these findings, it appears that the composition of the respiration buffer is perhaps the most critical factor in Ca^2+^ flux detection. In the multicomponent respiration medium (MiR05), Ca^2+^ fluxes were undetectable, even in the absence of a chelator, but a mannitol- and sucrose-based respiration buffer (without chelator) made the Ca^2+^ fluxes readily distinguishable from the addition of CaCl_2_. It may well be that some components of MiR05, such as lactobionic acid, taurine or bovine serum albumin, bind Ca^2+^ directly, or they make mitochondria resistant to external Ca^2+^ stress through membrane stabilization (antioxidant or inhibition of fatty acid oxidation) rendering Ca^2+^ flux detection impossible.

Measuring the duration of stimulated Ca^2+^ influx or efflux after single Ca^2+^ injections instead of multiple additions of relatively low concentrations of Ca^2+^ (as in the case of the CRC method) may be an alternative option to reducing the interval between individual additions, thereby minimizing differences originating from multiple Ca^2+^ exposure. These differences include distinct alterations in the depolarization of the inner mitochondrial membrane that may occur before mPTP opening. Not only can duration of fluxes be detected after a single Ca^2+^ injection, but also changes in fluorescence; thus, two parameters, time (*t*, *t1* and *t2* in s and their rate *t2*/*t1*) and relative fluorescent changes in intensity of Ca^2+^ indicator (∆*F*/*F*), can be evaluated at the same time and compared to the inhibitory effect of CsA. Further investigations are necessary to optimize the concentration of exogenous Ca^2+^ and specifically to determine whether more or less than 50 µM should be used to open the mPTP in isolated mitochondria in organs other than the liver. Based on our data, we suggest that simultaneous measurement of Ca^2+^-triggered Ca^2+^ and O_2_ flux may aid in testing and comparing (I) the effect of novel mPTP blockers (N-Me-Ala-6-cyclosporin A, N-Me-Val-4-cyclosporin and Sanglifehrin A^21^), which lack immunosuppressive effects, and (II) biologically active gases that are known to inhibit these channels, such as nitric oxide^22^, hydrogen sulfide^23^, isoflurane^24^, sevoflurane^25^ and noble gases^26^. Since mPTP-related fluxes were similar in isolated mitochondria and homogenate (both in the presence and absence of CsA), a time-consuming mitochondrial isolation procedure with low-speed and multiple high-speed centrifugation can be replaced by the use of homogenate, at least in the liver. Tissue homogenate provides numerous advantages over isolated mitochondria; for example, (I) the preparation protocol is faster; (II) tissue heterogeneity is well-preserved; and (III) a small amount of tissue is required for functional mitochondrial studies.

The question arises: how does uptake by mitochondria of external Ca^2+^ in the respiration buffer occur, and, secondly, what are the channels through which this Ca^2+^ can leave the organelle? Since our primary aim was to investigate the methodological aspects of Ca^2+^ fluxes, further experiments are needed to clarify the precise mechanism of influx and efflux in this model. A number of mitochondrial Ca^2+^ influx mechanisms have recently been identified in the literature, for instance, (I) rapid uptake mechanism-related Ca^2+^ uptake (RaM; quick, nanomolar Ca^2+^ influx^27^); (II) mitochondrial Ca^2+^ uniporter (which operates at a higher micromolar concentration of Ca^2+^^28^); (III) mitochondrial N-methyl-D-aspartate receptors^29^ in rat heart; (IV) leucine zipper-EF-hand containing transmembrane protein 1 (LETM1; the mitochondrial Ca^2+^/H^+^ antiporter^30^); (V) uncoupling protein (UCP^31^) 2 and 3; (VI) mitochondrial ryanodine receptor (mRyR^32^); and (VII) electron transport system-mediated Ca^2+^ uptake (coenzyme Q, which binds and transports Ca^2+^^33^).

As concerns efflux, so far three mechanisms have been documented in Ca^2+^ transport: (I) mPTPs^8^, (II) mitochondrial Na^+^/Ca^2+^ exchanger (mNCX^34^) and (III) Ca^2+^-proton (H^+^) exchanger (mHCX)^35^. Among Ca^2+^ entry mechanisms, the mitochondrial Ca^2+^ uniporter (MCU) seems to be a candidate since it operates at micromolar Ca^2+^ range (˃10 µM^36^), and the duration of Ca^2+^ uptake is slower than RaM-mediated influx (~ns). According to a potential model, it is suggested that Ca^2+^ transport through MCU is inhibited through MICU1 and MICU2 regulatory protein (“gatekeepers”) at a lower concentration of Ca^2+^, whereas it allows Ca^2+^ entry into the matrix at a higher concentration of Ca^2+^ as a result of conformational change in the protein via Ca^2+^ binding of EF hands of MICU1/MICU2^36^. Moreover, activation of mPTPs has been shown to be inhibited in MCU knockout mitochondria or after channel blockade with Ru360^37–40^. We hypothesize that RAM mechanism-mediated Ca^2+^ entry plays a negligible role or is undetectable in our measurements because changes in CaGreen-5N fluorescence occur at a higher (0.5–50 µM) concentration of Ca^2+^, whereas RAM operates at nanomolar concentration range^41,42^.

An increase in the level of intracellular Ca^2+^ can influence mitochondrial ROS formation^43^. Despite dissipation of mitochondrial membrane potential and mPTP activation, 50 µM CaCl_2_ did not affect the rate of extramitochondrial H_2_O_2_ generation in pilot studies using liver-isolated mitochondria (Supplementary Fig. S6). Substrate dependence of the effect of Ca^2+^ on ROS production has previously been described in the literature^43^. Thus, it may well be that other respiratory substrates should be added to succinate (or completely replaced) to stimulate the fatty acid oxidation pathway control state with octanoylcarnitine, palmitoylcarnitine (F-pathway) or the N-pathway with glutamate, malate and pyruvate to measure Ca^2+^-induced mitochondrial ROS generation.

In contrast with exogenous Ca^2+^-triggered Ca^2+^ flux, anoxia-related endogenous Ca^2+^ release occurred in all the types of samples studied. A continuous increase in CaGreen-5N fluorescent intensity was started immediately after the commencement of anoxia, with no decrease in its intensity after 30 min. The mechanism behind anoxic Ca^2+^ release may involve the possibilities noted above, i.e. mNCX, mHCX or mPTPs, or their combined action. In addition, responsiveness of fluorophore to exogenous CaCl_2_ was detected, and then the signal was successfully abolished with EGTA several hours (~3–4 h) later under anoxia (Fig. 5b). This observation facilitates the design of experiments with prolonged protocols and allows for a deeper insight into anoxia-induced mitochondrial Ca^2+^ movements and non-mitochondrial Ca^2+^ transport as well.

The study design has certain limitation because it is solely based on the High-Resolution FluoRespirometry technique. Besides, organelle-targeted biosensors can also be used to confirm the correlation between respiration activity and Ca^2+^ homeostasis in the cells. It should be noted here that metabolic differences may also affect the results. In isolated liver mitochondria and homogenate samples OXPHOS seems to be the predominant source of energy production while immortalized cell lines may privilege glycolysis over OXPHOS (Crabtree effect and Warburg effect). The different pathways for ATP production would largely affect mitochondrial function, including membrane potential and Ca^2+^ transport as well.

In summary, the new method is suitable to monitor simultaneous O_2_ and Ca^2+^ fluxes and the opening of mPTPs in various biological samples (in isolated mitochondria and tissue homogenate) after stimulation with external Ca^2+^. It can also be used to monitor anoxia-induced changes in Ca^2+^ release. Measuring the duration of stimulated Ca^2+^ fluxes may provide a novel parameter to evaluate the efficacy of mPTP blocker compounds.

## Supplementary information

Supplementary infornation
