# Supplementary material for: Ca(2+)N It Be Measured? Detection of Extramitochondrial Calcium Movement With High-Resolution FluoRespirometry
Source: Sci Rep. 2019 Dec 17;9:19229. doi: 10.1038/s41598-019-55618-5 (PMC6917783; doi:10.1038/s41598-019-55618-5)
Supplement: Supplementary file 1 — Supplementary infornation [file 41598_2019_55618_MOESM1_ESM.pdf]

$Ca^{(2+)}$N It Be Measured? Detection of Extramitochondrial Calcium Movement With High-Resolution FluoRespirometry

Anna Nászai, Emil Terhes, József Kaszaki, Mihály Boros, László Juhász

University of Szeged, Faculty of Medicine, Institute of Surgical Research, Szeged, Hungary

Corresponding author: László Juhász

Institute of Surgical Research, University of Szeged

e-mail: juhasz.laszlo.1@med.u-szeged.hu

SUPPLEMENTARY FIGURES

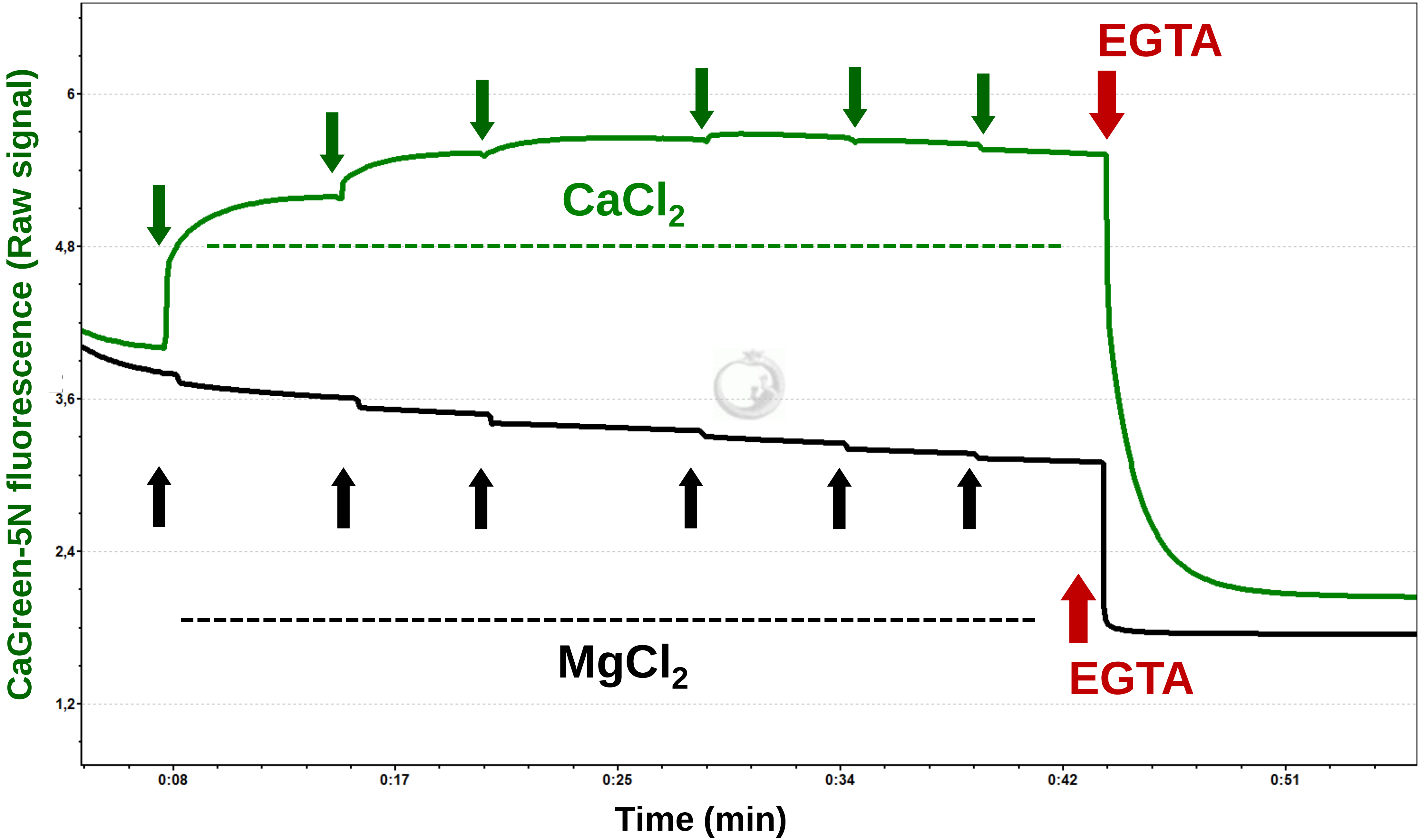

Supplementary Figure S1

Supplementary Figure S1. The effect of repeated addition of $MgCl_2$ on CaGreen-5N fluorescence. Arrows illustrate a single administration of 50 μM $MgCl_2$ (black), 50 μM $CaCl_2$ (green) and 1 mM EGTA (red). Green and black lines illustrate time course changes in CaGreen-5N fluorescence. Measurements were performed in 2 mL mannitol- and sucrose-based medium at 37ºC.

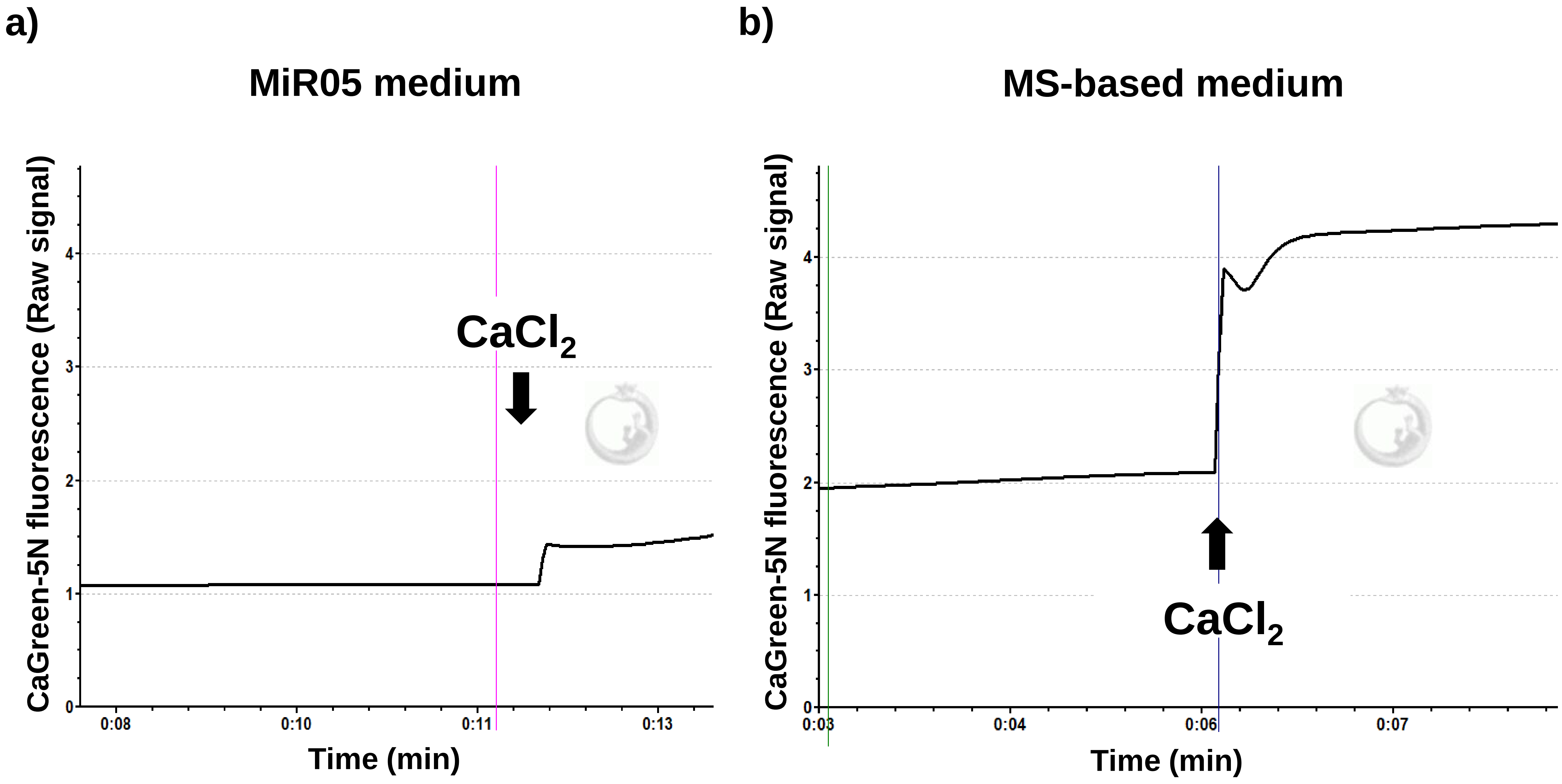

Supplementary Figure S2

Supplementary Figure S2. Ca^{2+} fluxes in MiR05 medium without 0.5 mM EGTA (a) and in a mannitol- and sucrose (MS)-based (b) respiration buffer. Stimulation with 50 µM $CaCl_2$ increased fluorescence in both media; however, Ca^{2+} fluxes could only be detected in the MS-based buffer.

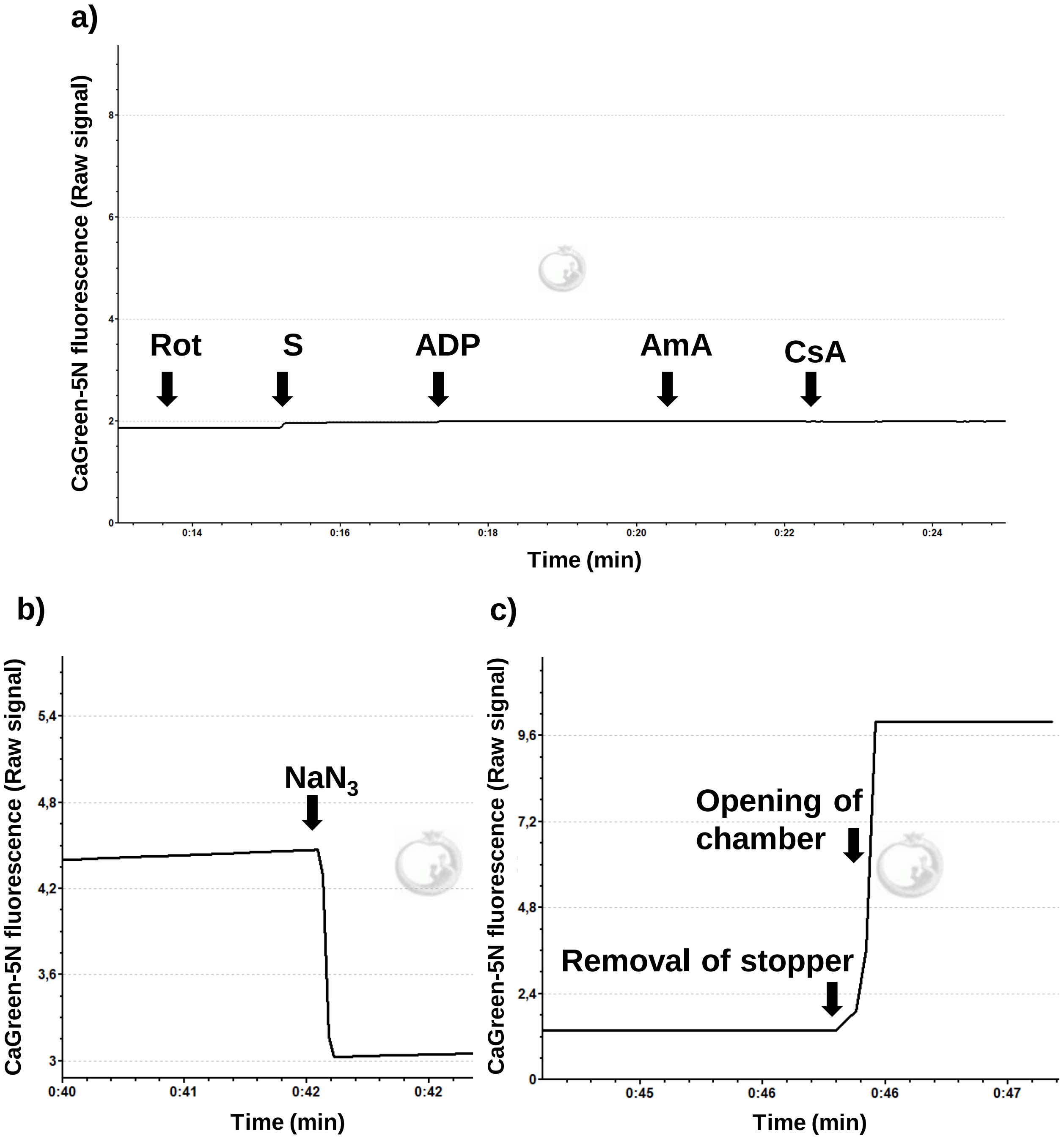

Supplementary Figure S3

Supplementary Figure S3. The effect of respiratory substrate/inhibitors (a) on CaGreen-5N fluorescence and artefacts (b and c) using CaGreen-5N indicator. Abbreviations: Rot: rotenone; S: succinate; ADP: adenosine diphosphate; AmA: antimycin A; CsA: cyclosporin A. Sodium azide (NaN_3; b) and chamber opening (c) affected CaGreen-5N signals, resulting in a fluorescent artefact. NaN_3 decreased, whereas the opening of the respiration chambers increased fluorescence.

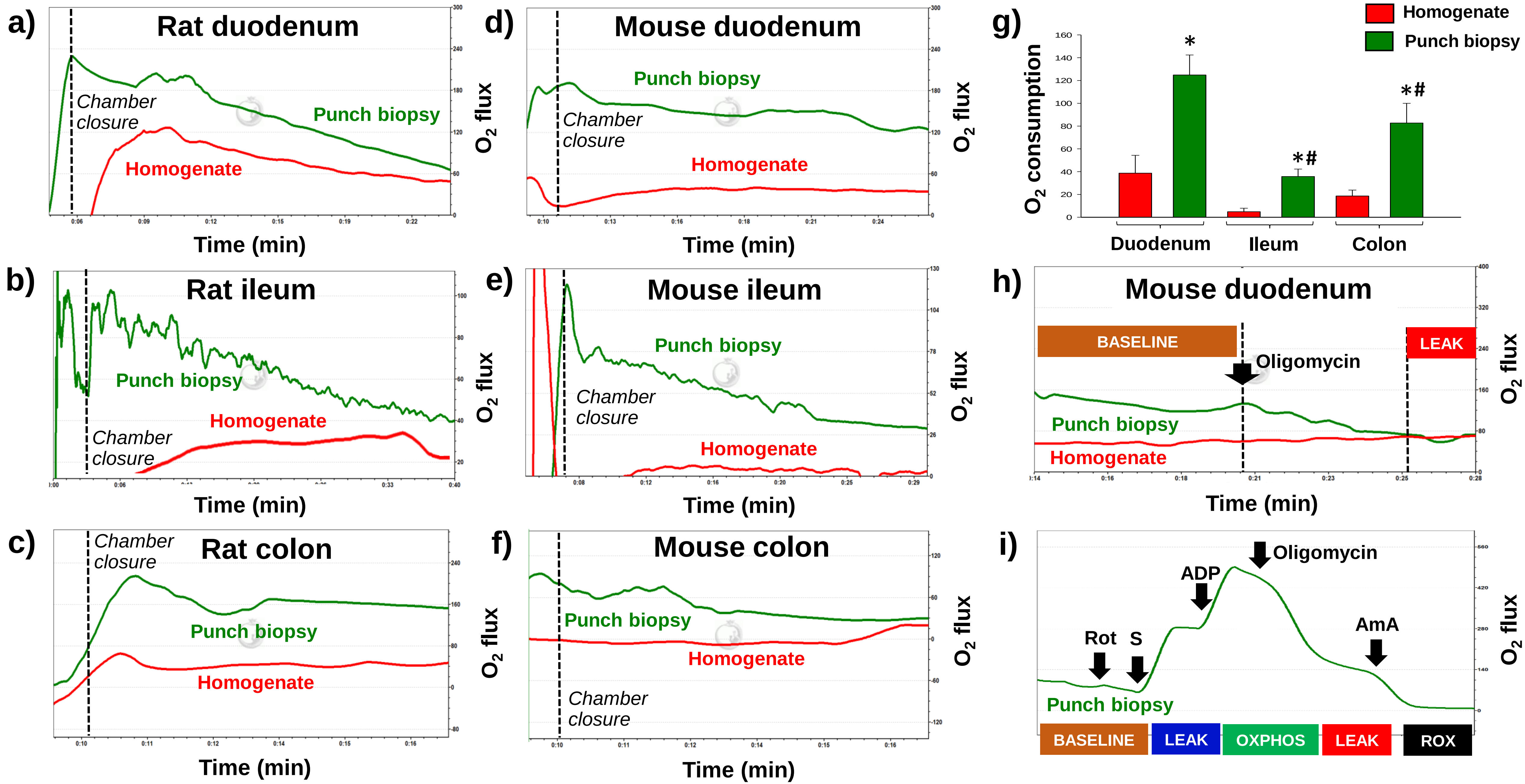

Supplementary Figure S4

Supplementary Figure S4. Representative traces obtained from SPRD rats and SKH-1 mice illustrating O_2 consumption by intestinal punch biopsies and homogenates (a–i). Lower baseline respiration was registered in homogenates than in punch biopsies. Stable baseline O_2 flux (without external substrate and ADP) was obtained from rat colon and from mouse duodenum and colon samples. Among them, mouse duodenum showed the highest baseline O_2 flux (Figure 4g), and they were responsive for ATP synthase inhibitor, with or without stimulation of OXPHOS (Figure 4h and 4i). Data were expressed as mean+SD; One-way ANOVA; *P<0.05 Homogenate vs Punch and # vs Duodenum Punch (n=4). Measurements were performed in Mir05 medium at 37°C.

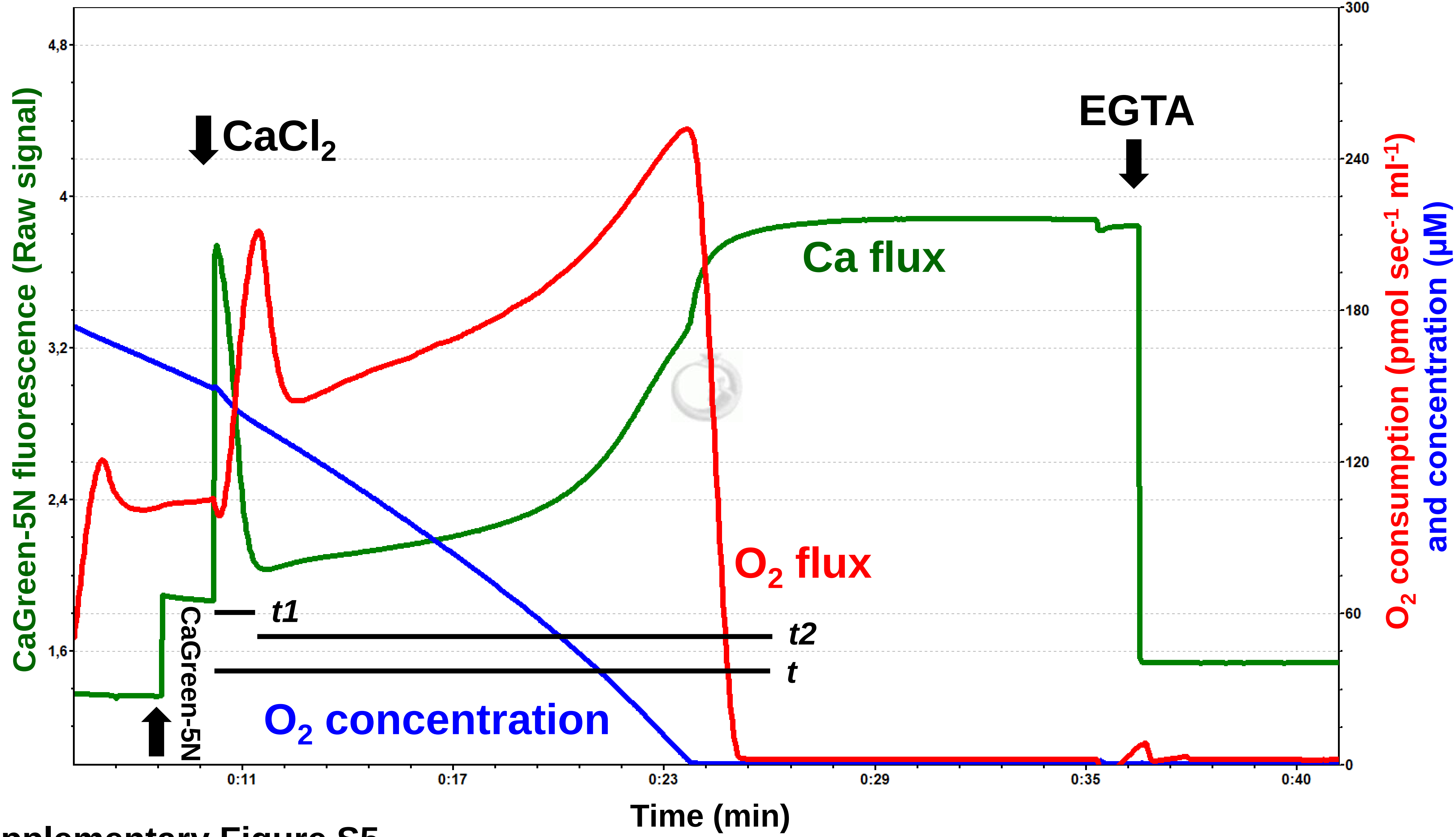

Supplementary Figure S5

Supplementary Figure S5. Simultaneous measurement of Ca^{2+} and O_2 flux after pre-incubation with CsA in SKH-1 mouse liver homogenate. Inhibition of mPTPs by cyclosporin A (CsA)-elevated Ca^{2+} uptake and delayed Ca^{2+} efflux after stimulation with exogenous Ca^{2+}. Superimposed lines show extramitochondrial Ca^{2+} flux (green), mitochondrial O_2 consumption (O_2 flux; red) and O_2 concentration (blue). Black lines indicate the duration of Ca^{2+} fluxes (*t*), and the component influx (*t1*) and efflux (*t2*).

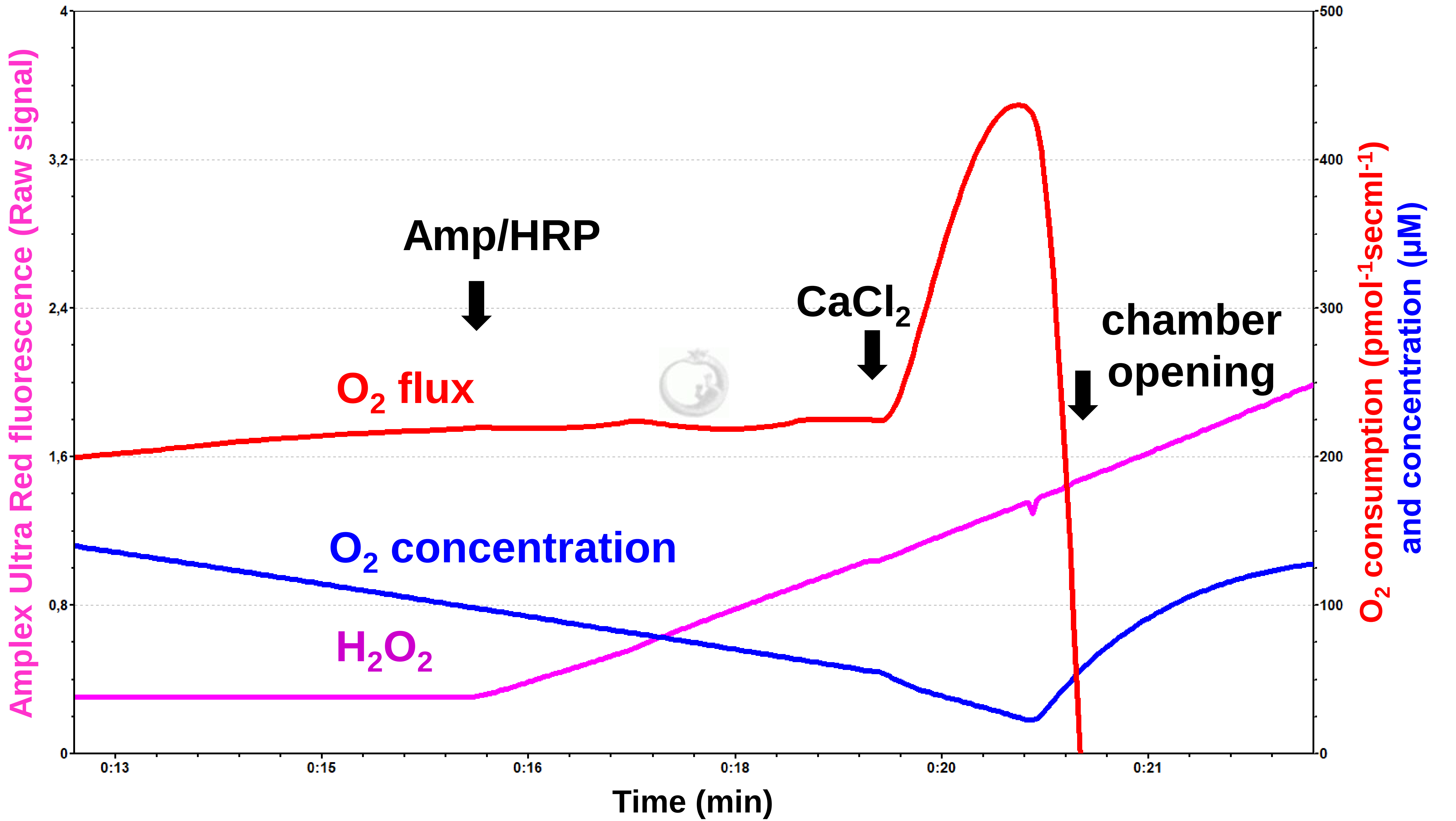

Supplementary Figure S6

Supplementary Figure S6. The effect of 50 µM $CaCl_2$ on H_2O_2 generation and O_2 flux in rat isolated liver mitochondria. After respiration was stabilized, Amplex Ultra Red (Amp) and horseradish peroxidase (HRP) were injected into energized mitochondria to assess H_2O_2 production (resorufin). A continuous increase in the level of extramitochondrial H_2O_2 was observed, which was not affected by the addition of Ca^{2+}. Superimposed lines illustrate extramitochondrial H_2O_2 production (pink), mitochondrial O_2 consumption (O_2 flux; red) and O_2 concentration (blue).
